# Letter from Professor Martins

**Published:** 1840-05-23

**Authors:** 


### FOREIGN CORRESPONDENCE.

### LETTER FROM PROFESSOR MARTINS. No. VII.

*Proceedings of the Societé Médicale d'Observation—Meeting of the* 14*th March*, 1840—*M. Louis, the President, in the chair.*

To the Editors of the Medical Examiner.

M. Legendre, interne at the hospital *La Pitié*, read the following case of

*Pleurisy of the Right Side, followed by Hydro-Pneumothorax.*

A man named Lebrouissard, aged thirty-seven years, a sausage-seller, born at Le Mans, entered La Pitié the 5th September, 1839. He is a man five feet three inches in height, of chesnut hair, hazel eyes, and considerably emaciated in his limbs. The last phalanx of every finger is large, with the nail curved in, so that the finger terminates like a club. According to the patient, this deformity has existed only since the attack of the disease. He has considerable intelligence, and gives a very good account of himself previous to his entrance into La Pitié. His mother was a stout and healthy woman, who fell a victim to some epidemic; his father is still alive, and, although sixty-six years of age, works as a day-labourer. He has two sisters, likewise healthy. He was vaccinated in infancy, and does not remember to have had the measles. He was, however, up to the age of ten or twelve, subject to eruptions on the hairy scalp, which produced enlargements of the cervical and submaxillary ganglia, which terminated by resolution. At fifteen years of age he came to Paris, and was put apprentice to a sausage-maker. His health was always excellent; he committed neither alcoholic nor venereal excesses, drinking only about three or four small glasses of brandy every morning. A hard worker, he often exposed himself to the cold while in a perspiration, but was not particularly subject to colds during the winter. His respiration was long, and he could run a considerable distance without being out of breath.

In the month of December, 1836, he was seized for four days with irregular chills, followed by smart flushings, and he does not remember to have suffered from a stitch in the side. The fourth day, he was copiously bled twice in the day; the next morning a considerable number of leeches were applied to the epigastric region. After the bleeding he began to cough; and the attacks of coughing gave him great pain in the breast, especially on the right side. The physicians called at the onset of the attack, ausculted him, and pronounced him to be labouring under a pleuro-pneumony of the right side. The loss of blood had singularly weakened the patient; he remained three months at his master's, without completely recovering. He coughed constantly, and expectorated opaque and greenish sputa. He was treated merely by lozenges and demulcent drinks. He then determined to return to his native country, whither he transported himself by the diligence, and, when arrived, remained in bed for four months longer. At this time he noticed that the right side of his breast projected more than the other. During the last two months of his confinement to bed, he was put upon the use of the juice of cresses, in the dose of two spoonfuls a day, roast meat and wine. When he began to rise, he was each time taken with a violent fit of coughing, with copious expectoration of thick and greenish sputa. He never spit any blood. Milk was at this time his only nourishment, which he digested with difficulty, without, however, rejecting it from his stomach. He had no continuous diarrhœa during this whole period. Fifteen months before his entrance into the hospital, about June,

1838, his sputa became all at once fœtid, without this phenomenon's being accompanied by any new morbid symptoms. This fœtidity was constant. Five months after, the right side of the breast began to show gradually diminution in size. The patient, in whose condition there was no change, decided to return to Paris in the month of January, 1839. He entered first the hospital La Charité, in the wards of M. Andral, where he remained for five weeks under the use of common tisan. He then passed under the care of M. Louis, at the hospital Beaujon; but the fœtidity of his sputa was such as to incommode all the patients in the same ward, which was small, as is the case with all the wards of this hospital. L. was transferred to the hospital La Pitié, under the care of M. Mailly.

The 8th of October, 1839, his condition was as follows:—Limbs emaciated; no strength; skin earthy, a little dry, without being hot; no sweats. Pulse eighty, regular; no palpitations. Tongue moist, rosy; appetite good; no diarrhœa. He has, however, a sensation of pain shooting across the abdomen, below the umbilicus. So long as the patient is in bed, he has no cough; but as soon as he rises, he is taken with cough and copious expectoration. The cough is cavernous, loud; the sputa opaque, greenish, irregular in form, and floating in a syrupy and transparent liquid. Their odour presents curious alternations: sometimes they exhale a very fœtid, gangrenous-like smell, which disappears, and again immediately returns. The same may be said of his breath. This fœtidity is connected with an amphoric respiration, which disappears with it, but constantly accompanies it. The metallic tinkling has never been heard. The patient thinks he can perceive that the sputa come from the right inferior side of the breast; there seems to him to exist there a sort of reservoir, which by turns fills and empties itself by expectoration, morning and evening. The cavity once empty, the patient is tranquil. During the month of August, M. Louis estimated at two pounds and a half the sputa expectorated during twenty-four hours; it would now take him three days to throw up the same quantity. The breast presents a very visible sinking in, on the right side. On this side, the breast is nine lines less in circumference than on the opposite side. Percussion is sonorous on the left side; the respiration is pure, the beatings and sounds of the heart normal. On the right the percussion is sonorous to the extent of four fingers' breadth below the clavicle; below this point flatness commences, and extends below. A slight respiratory murmur is heard throughout the sonorous region. Posteriorly, on the right, flatness and absence of the respiratory murmur; no modification in the auscultation of the voice, neither resonance nor bronchophony. At the moment of examination, the sputa not being fœtid, there is no amphoric respiration, nor rhonchus, nor gurgling. Prescription—pectoral ptisan, julap of syrup of poppies, fumigations with six drops of chlorine.

*10th and 11th of October.*—Amphoric respiration in the two inferior thirds of the breast, mixed with a little mucous rhonchus; fœtidity of the breath and sputa. On the 11th, the mucous rhonchus is transformed into a true gurgling.

*16th.* Amphoric respiration and fœtidity of the breath and sputa continue; but are much less marked.

*18th.*—Discontinuance of the amphoric respiration, and of the mucous rhonchus; the breath and sputa no longer fœtid.

*20th* to *26th.*—The amphoric respiration has reappeared, accompanied by fœtidity of the sputa.

*30th.*—The patient is seized with suffocation so as to endanger his life. Sinapisms to the soles of his feet; blisters to the thighs.

*4th of November.* The attack of dyspnœa has not returned; but, from this period, the patient is weak and without appetite; he scarcely eats at all, and his chest presents alternations of amphoric respiration and absence of all abnormal sounds.

*15th.*—For the last four or five days, the patient has experienced momentary attacks of dyspnœa, without, however, being forced to sit up; he does not expectorate the same quantity, the sputa are no longer fœtid, and the amphoric respiration has disappeared. Pulse seventy, regular; appetite none. Six wet cups to the base of the sternum.

*23d.*—In the night the patient suddenly expired, without having offered any other remarkable phenomena, preserving consciousness to the last moment. From the 15th of November his condition had been the following: Little fever, paroxysms of dyspnœa from time to time. The cough had nearly ceased, and the expectoration was insignificant. The breath and sputa were without smell; no amphoric respiration; weakness so great that the patient was obliged to be assisted in sitting up for auscultation. Complete loss of appetite; no derangement of the bowels.

*Autopsy*, the 25th of November, in the morning. No appearance of decomposition; cadaveric rigidity pretty well marked. The right side of the chest evidently sunken in. The organs of digestion, the brain, and the bladder, do not offer the slightest trace of alteration.

*Chest.* The *left* lung adheres throughout closely to the costal pleura by a very compact (but not fibrous) cellular tissue. The lung on this side is soft, crepitates, but has scattered throughout it a multitude of gray granulations, semi-transparent, of the diameter of a millet-seed. Towards the summit are found two tubercles of the size of a hemp-seed, white and friable, apparently having a tendency to pass into the cretaceous state. This pulmonary tissue floats readily, and contains no cavity.

The *right* lung occupies the superior portion of the thoracic cavity. It is only five or six inches in height. At least a half litre of viscid, thick, greenish, fœtid pus, almost completely fills a cavity, the walls of which are formed, above, by the base of the right lung, below by the walls of the chest and by the mediastinum. The walls of this cavity are covered by a membrane at least a line thick, grayish, very dense and resisting, in a word, of a fibrinous character. In some points, this false membrane is covered with thin, soft, pseudo-membranous coatings; at other points are seen irregular calcareous concretions, three or four lines in diameter. Finally, towards the internal portion of the base of the lung, the pseudo-membranous coating passes into the fibrous state. Instead of being grayish, as at the other points, it is of a deep red colour. At the centre of this surface so coloured, are three little openings, from half a line to a line in diameter, which lead to a bronchia five lines in circumference. The internal surface of this bronchia is of a deep red, and has a grainy structure. The mucous membrane appears a little thickened, without, however, being softened. Immediately above the fibrous membrane which supports the base of the lung, the pulmonary tissue is condensed, is of a greenish colour, and exhales a fœtid odour. Its cohesion is very great, and this circumstance is incompatible with all idea of a gangrene of the lung; it floats very readily. The indurated tissue occupies a height of about an inch of the base of the lung; above, the parenchyma is soft, suple, and crepitating. It is only at the summit, that are found gray semi-transparent granulations, of which some, the size of a hemp-seed, offer a white colour in their centre. In fine, these granulations are less numerous than on the other side, and none are observed at the base. In blowing into the trachea, the purulent liquid contained in the pleural cavity was caused to bubble. The ribs of the right side were thicker than those of the left—about seven or eight lines in diameter.

*Remarks by the author*, (M. Legendre.)—The habitual good health of the patient, the absence of hemoptysis and of other symptoms as regards the respiratory apparatus up to the period of the attack of pleuropneumony on the right side, three years back; the progress of this affection, the increased and afterwards the strikingly diminished size of the chest, coinciding with the fœtidity of the sputa; the absence of physical signs of tubercles on the left side of the chest, and even on the right side under the clavicle—would all induce me to suppose that this hydro-pneumothorax was the result, not of a tuberculous ulceration of the pulmonary tissue, which terminated in effecting a communication between the external air and the pleuritic effusion. The autopsy, I think, has confirmed this opinion. For, in fact, in the midst of a crepitating pulmonary tissue, are found only gray semi-transparent granulations, and a very small number of tubercles in a crude state. These alterations were even in smaller numbers on the right than left side, and occupied only the summit of the lung, whilst, at the base, in the situation of the fistula, there was not the most minute granulation. There are other symptoms which demand our attention. These are, first, the alternations of disappearance and return of the amphoric respiration, which was always connected with a fœtidity of breath and sputa. This fœtidity never disappeared without a similar disappearance of the amphoric respiration. These intermissions can be explained only by supposing alternations of obstruction in the pleuro-bronchial fistula. Secondly, is to be remarked the flatness, which was established by percussion, in place of the extreme sonorousness which usually accompanies hydro-pneumothorax. This difference may be explained by the long standing of the disease. Thus, at the onset of the perforation, the extreme sonorousness may have existed, then have gradually ceased, from the fact of the contracting of the abnormal cavity. Thirdly, we should notice the absence of the metallic tinkling, which, however, no attempt was made to elicit, by practising the hippocratic succussion.

*Debate on the preceding case.*

M. Fauvel would be glad to have some details respecting the calcareous concretions found in the pleural cavity. He did not agree with the opinion of M. Legendre, but considered the pleuro-bronchial fistula as the result of a tuberculous ulceration. The frequency of ulcerations of this character, the extreme rareness of a simple ulceration, besides the existence of tubercles in the midst of the pulmonary tissue, he deemed sufficient reasons to reject the opinion put forth by the author.

M. Legendre, in reply, stated, that the calcareous concretions were irregular, and were found upon the free surface of the false membrane which lined the costal pleura. He could not relinquish his view of the cause of the perforation.

M. Voillemier was anxious for further details touching the increase followed by decrease in the volume of the chest, the sputa, and the pathological anatomy of the fistulous passage.

M. Legendre replied that he had not witnessed the changes in the volume of the chest, but had gathered them from the statement of the patient; he re-read the portion of the case where the sputa and fistulous passage are mentioned.

Dr. Barth wished for some details respecting the cough, and the mode in which the sputa were expectorated; he regretted that the author had not stated whether the flatness were complete or incomplete, that he had not compared posteriorly the results of percussion in the right and left supra and infra-spinal fossæ, nor indicated the point where the amphoric respiration showed its maximum of intensity. He agreed with M. Fauvel with regard to the cause of the perforation of the pulmonary tissue.

M. Legendre replied that the flatness was nearly absolute; it was under the inferior angle of the shoulder-blade that the amphoric respiration was best heard.

Dr. Valleix asked if the dyspnœa was continuous or paroxysmal, and whether a false membrane lined the mucous membrane of the fistulous passage: he agreed with the opinions expressed by MM. Fauvel and Barth.

M. Legendre: The dyspnœa, which was marked only in the latter stage of the disease, was paroxysmal; there was no false membrane lining the bronchial mucous membrane.

Dr. Grisolles adverted to the omission to state whether œdema existed in the right thoracic paries, and whether the fistulous opening was furnished with any kind of valve, as has been noticed in some analogous cases. Dr. Grisolles, while taking the same side of the question as MM. Barth, Fauvel, and Valleix, admitted that there was one circumstance in favour of the opinion of M. Legendre—the absence of the sudden, instantaneous symptoms which accompany a perforation of the lung, the result of tubercle.

M. Legendre: There was neither œdema nor appearance of valves. I closely interrogated the patient for the purpose of ascertaining, if, at any period, he had experienced any sudden aggravation of his symptoms, and he invariably replied in the negative.

M. Fleury, formerly interne of M. Louis, who had noted the case at the hospital Beaujon, confirmed the principal facts stated by M. Legendre. He had never heard metallic tinkling.

Dr. Barth remarked, that the position of the fistula, in relation to the liquid, might explain the absence of this abnormal sound.

Dr. Louis said, that, in defence of his opinion as to the perforation of the lung, M. Legendre had dwelt upon the absence of an intermediate cavity, attributable to the softening of a tubercle, between the bronchia and the fistulous opening. This cavity, Dr. Louis observed, does not always exist in cases of tubercular perforation. Dr. Louis coincided with the opinion expressed by MM. Fauvel, Barth, Grisolle, and Valleix, respecting the origin of this perforation.

